# Ion channel-mediated mitochondrial volume regulation and its relationship with mitochondrial dynamics

**DOI:** 10.1080/19336950.2024.2335467

**Published:** 2024-03-28

**Authors:** Yujia Zhuang, Wenting Jiang, Zhe Zhao, Wencui Li, Zhiqin Deng, Jianquan Liu

**Affiliations:** a Hand and Foot Surgery Department, Shenzhen Second People’s Hospital/the First Hospital Affiliated to Shenzhen University, Shenzhen, China; b Clinical College of Shantou University Medical College, Shantou, China; c Operating room, Shenzhen Second People’s Hospital/the First Hospital Affiliated to Shenzhen University, Shenzhen, China

**Keywords:** Ion channels, mitochondrial, cell volume regulation

## Abstract

The mitochondrion, one of the important cellular organelles, has the major function of generating adenosine triphosphate and plays an important role in maintaining cellular homeostasis, governing signal transduction, regulating membrane potential, controlling programmed cell death and modulating cell proliferation. The dynamic balance of mitochondrial volume is an important factor required for maintaining the structural integrity of the organelle and exerting corresponding functions. Changes in the mitochondrial volume are closely reflected in a series of biological functions and pathological changes. The mitochondrial volume is controlled by the osmotic balance between the cytoplasm and the mitochondrial matrix. Thus, any disruption in the influx of the main ion, potassium, into the cells can disturb the osmotic balance between the cytoplasm and the matrix, leading to water movement between these compartments and subsequent alterations in mitochondrial volume. Recent studies have shown that mitochondrial volume homeostasis is closely implicated in a variety of diseases. In this review, we provide an overview of the main influencing factors and research progress in the field of mitochondrial volume homeostasis.

## GRAPHICAL ABSTRACT

**Figure:**
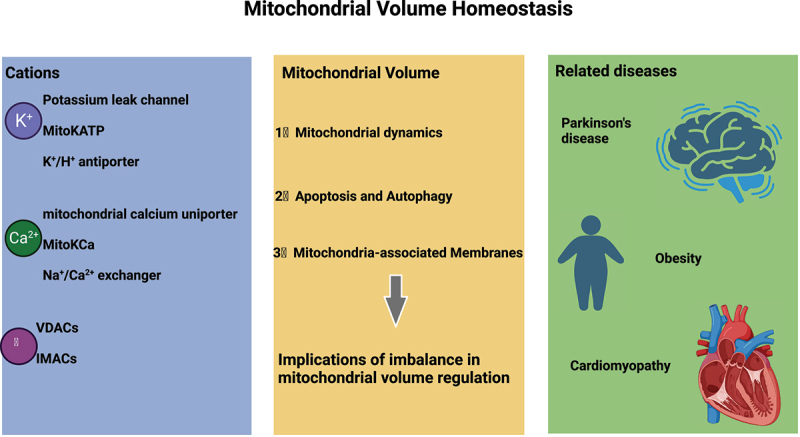

## Introduction

In a eukaryotic cell, the mitochondrion is the central site for oxidative metabolism, a process wherein sugars, fats and amino acids are finally oxidized to release energy. The mitochondrion also participates in the dynamic regulation of calcium ion concentration, cell proliferation and differentiation, cellular metabolism, signal transduction, autophagy and apoptosis. Maintenance of the normal morphology and function of the mitochondrion is an important requirement for the organelle to carry out its various physiological activities in the cell. Under diverse stress conditions, the mitochondrion exhibits heightened vulnerability to damage and dysfunction, intricately tied to the emergence of a multitude of diseases, including cardiovascular ailments, neurodegenerative disorders, metabolic irregularities, and visual impairments [1]. Mitochondrial volume homeostasis is a dynamic regulatory process mainly controlled by the cation channels located on the inner mitochondrial membrane. An imbalance in the mitochondrial volume homeostasis will affect a series of cellular functions. We herein present a review that summarizes the main factors influencing mitochondrial volume homeostasis.

## Mitochondrial volume homeostasis

### Mechanism of mitochondrial volume homeostasis

The mitochondrion, a double-membraned organelle, exerts its cellular functions in an intracellular environment rich in ions such as Na^+^, K^+^ and Ca^2+^ [2]. Early studies have shown that the inner mitochondrial membrane is impermeable to these ions, and their concentrations in the mitochondrial matrix are regulated by specific channels and transporters [2]; however, the inner membrane is highly permeable to water [3]. This property makes the mitochondrion highly sensitive to changes in osmotic pressure in the surrounding environment, thereby promoting and maintaining the osmotic balance between the mitochondrial matrix and the cytoplasm. In 1915, Lewis et al. [4] reported that the mitochondrial morphology in cultured chicken embryo cells showed high plasticity and that the mitochondrion could reversibly expand and contract with changes in the pH or osmotic pressure of the culture medium. Therefore, when ion channels distributed within mitochondria are stimulated by signals, their function undergoes changes (opening or closing). The movement of ions across the mitochondrial membrane alters the osmotic pressure equilibrium on both sides, facilitating the ingress and egress of water and resulting in volume fluctuations within a specific range. This process is called the dynamic balance or volume homeostasis of the mitochondrion.

### Effect of mitochondrial volume changes on cell function

Mitochondrial volume homeostasis not only plays an important role in maintaining the structural integrity of the organelle but also affects multiple physiological functions of the cell. Halestrap et al. [5–7] reported that mitochondrial volume is closely related to the cellular respiration rate, wherein an increase in matrix volume can activate the mitochondrial respiratory chain, thereby increasing ATP production. Some studies have shown that the dynamic equilibrium of mitochondrial volume is closely related to the formation of reactive oxygen species (ROS) [8]. An increase in K^+^ influx can increase mitochondrial volume, leading to matrix alkalinization [9], mild mitochondrial uncoupling [10]and enhanced fatty acid oxidation [5], all of which can promote the release of ROS. Gogvadze et al. [11] pointed out that changes in mitochondrial volume during cell apoptosis may play an important role in regulating the release of cytochrome c. Kaasik et al. [12]reported that an increase in mitochondrial volume in cardiomyocytes can impose mechanical constraints within the cell, resulting in increasing contractile force of the myofibrils (Table 1).

**Table 1.** Mitochondrial ion channels described in this article.

	Type	Role(s)	Selected refs.
1	K_ATP_	Volume regulation, protection against apoptosis/ischemic injury	[16,22]
2	K+ leak	Volume regulation	[14]
3	K+/H+ antiporter	Volume regulation	[26,27]
4	Ca^2+^ uniporter	Ca^2+^ uptake	[31]
5	K_Ca_	Volume regulation	[32–34]
6	VDAC	Metabolite transport, Cytochrome c release/apoptosis, PTP complex	[38]
7	IMACs	Volume regulation	[34,40]
8	ClIC4,5	Volume regulation,apoptosis	[43]

## Cations regulate mitochondrial volume homeostasis

### Effect of potassium ions on mitochondrial volume

**Figure 1.**
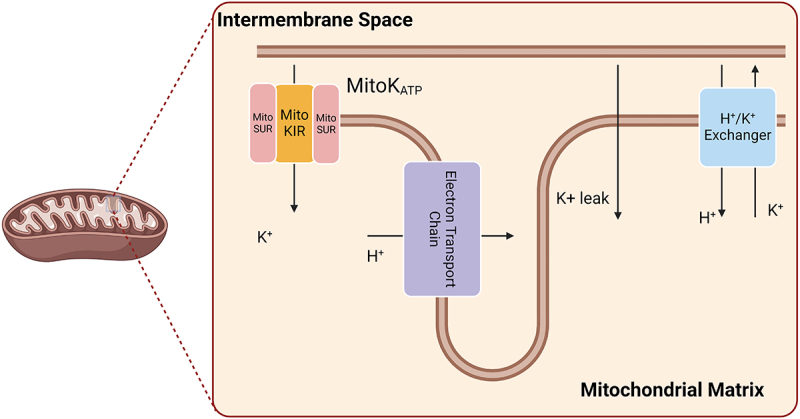
Overview of mitochondrial K^+^ transport.

The intracellular concentration of potassium ions is significantly higher than that of other ions; therefore, the osmotic equilibrium between the cytoplasm and the mitochondrial matrix is believed to be mainly controlled by potassium ion flux, and potassium homeostasis is considered the main regulatory factor for mitochondrial matrix volume [2]. Since 1961, which was when Mitchell [13] proposed the chemiosmotic theory, K^+^ cycling in the mitochondrion has gradually gained recognition; this cycling involves the influx and efflux of K^+^, H^+^ and anions, mainly carried out through ATP-sensitive potassium channels, potassium leak channels and H^+^/K^+^ exchange. This cycling has been proven to be crucial for certain cellular phenomena including the regulation of mitochondrial volume and redox reactions [14] (Figure 1).

Because of proton pumping by the electron transport chain (ETC) coupled with the reduction of oxygen and oxidation of coenzymes, the mitochondrion has an electrochemical gradient, with a negatively charged matrix. K^+^ leak across the inner membrane is quantitatively relevant owing to the high concentrations of this ion in the cytosol and the electrochemical gradient. K^+^ ions also enter the matrix through the ATP-sensitive K^+^ channel (KATP or MitoKATP). The channel is formed by two subunits: MitoKIR (or MitoK) and MitoSUR. K^+^ is removed from the mitochondrial matrix in exchange for H^+^

#### Potassium leak channel

In a eukaryotic cell, the sites for the electron transport chain and oxidative phosphorylation are located on the inner mitochondrial membrane. The release of energy resulting from the reaction of oxygen molecules with reducing compounds such as cytochrome c, NADH and FADH occurs through the electron transport chain, wherein protons are pumped into the intermembrane space, thereby creating an electrochemical gradient on the inner mitochondrial membrane with a negative inside. Because intracellular potassium ions concentrations are high, in the range of 140 mM, and the mitochondrial inner membrane has an electrochemical potential in the range of 100 to 200 mV, potassium ions are expected to diffusion(potassium ions leak) through the membrane at biologically relevant rates [14]. The potassium leak channel refers to the nonselective potassium channels located on the inner mitochondrial membrane. Unlike mitoKATP channels, potassium leak channels passively transport potassium ions down their electrochemical gradient without regulation by ATP or other factors. Although the mitochondrial membrane is very poorly permeable to these ions, cation diffusion occurs very rapidly, and they are important at the physiological level: K^+^ diffusion occurs inward through potassium leak channels and can lead to matrix swelling [15]. Because the inward diffusion of K^+^ ions through potassium leak channels depends on the mitochondrial membrane potential, it is closely related to the metabolic state of the cell.

#### ATP-sensitive potassium channels

In 1991, Inoue et al. discovered the presence of K^+^ channels on the inner mitochondrial membrane that are sensitive to ATP (MitoKATP) using patch-clamp techniques. Subsequently, Paucek et al. [16] isolated and reconstituted MitoKATP and found that the reconstituted sample of the mitochondrion contained two bands: a 55-kDa protein (MitoKIR), which acts as the K^+^ channel, and a 63-kDa protein (MitoSUR), which acts as a channel regulator. This channel has important physiological functions. First, it can maintain the dynamic balance of K^+^ ions in the mitochondrion, thereby controlling the mitochondrial volume [17]. Second, the uptake of K^+^ ions from the cytoplasm by the mitochondrion can partially compensate for the charge transfer generated by proton pumps, thereby forming pH gradients and transmembrane potentials [18]. In addition, MitoKATP plays an important role in regulating the release of mitochondrial ROS. Schumacher et al. [19] reported that, when cardiomyocytes are treated with MitoKATP agonists, the release of mitochondrial ROS is reduced.

MitoKATP is regulated by various biological factors. Unlike other KATP channels, MitoKATP is inhibited by both ATP and ADP but is activated by GTP and GDP. However, the ATP- or ADP-related inhibition requires the presence of Mg^2+^ [16], and MitoKATP is also physiologically inhibited by long-chain acyl-CoA esters [20]. MitoKATP is also controlled by kinases and activated by Protein Kinase C agonists [21]. This channel is also regulated by the activity of respiratory complex II and responds to endogenous complex II inhibitors such as malonate [22]. In addition to physiological regulation, the channel is regulated by various drugs including agonists such as diazoxide, nicorandil, BMS-191095 and pinacidil as well as antagonists such as 5-hydroxydecanoate and glibenclamide [23].

Activation of MitoKATP by various agonists described above leads to an influx of K^+^ ions into the mitochondrial matrix, coupled with anion transport(Pi^−^), which changes the osmotic pressure of the mitochondrion and facilitates the diffusion of water into the mitochondrial matrix, thus promoting mitochondrial swelling. This feature is important for maintaining normal mitochondrial structure and regulating the intermembrane space structure [3]. Therefore, MitoKATP activation may prevent pathological over-contraction from structurally damaging the mitochondrion and preserve transport characteristics of the mitochondrial membrane, including the transport of ADP and ATP [23].

#### K^+^/H^+^ antiporter

Garlid et al. [24–26] discovered and confirmed the presence of the K^+^/H^+^ antiporter through experimentation. The antiporter transports one unit of H^+^ ions into the mitochondrial matrix while exporting one unit of K^+^ ions out of the mitochondrion. The mitochondrial K^+^/H^+^ antiporter is reversibly inhibited by Mg^2+^ ions, protons and amphiphiles and irreversibly inhibited by dicyclohexylcarbodiimide [9]. In addition, some researchers have reported that even if Mg^2+^ ions are depleted in the mitochondrial matrix, increasing the matrix volume can activate K^+^/H^+^ counter-transport [27,28], which may be due to conformational changes caused by mitochondrial membrane stretching or interactions between the conformational center and other matrix solutes when Mg^2+^ and H^+^ ions decrease under non-physiological conditions.

K^+^ ions, which are the most abundant in the cytoplasm, play a predominant role in regulating mitochondrial volume. The two potassium channels – the potassium leak channel and MitoKATP channel, as well as the K^+^/H^+^ antiporter, constitute the mechanisms of potassium transport involved in the mitochondrial K^+^ cycle that regulates matrix volume. The electron transport chain on the inner mitochondrial membrane generates membrane potential by releasing protons, which drives the influx of K^+^ ions through K^+^ leak channels and MitoKATP. This exchange of K^+^ and H^+^ will increase the alkalinity of the matrix, leading to the entry of phosphate ions through the Pi-H^+^ antiporter, and the net uptake of K^+^ ions will be accompanied by water retention, resulting in matrix swelling. The matrix swelling subsequently activates K^+^/H^+^ antiporters, and excess K^+^ ions in the matrix are then expelled by the K^+^/H^+^ antiporter [9]. This cycle of K^+^ ions maintains mitochondrial volume at a steady state equilibrium.

### Effects of calcium ions on mitochondrial volume

The effects of Ca^2+^ ions on mitochondrial volume have also been widely studied, and the main calcium influx mechanisms include mitochondrial calcium uniporter (MCU), rapid uptake mode and mitochondrial ryanodine receptor. In 2011, De Stefani et al. [29] confirmed that Ca^2+^ ions mainly enter the mitochondrial matrix through a 40-kDa protein located on the inner mitochondrial membrane, namely, MCU. The MCU is the main calcium uptake channel that is critically dependent on the inner mitochondrial membrane potential (ΔΨm). Its activity is controlled by the mitochondrial calcium uptake 1 protein (MICU1), which is the basic regulatory factor for the uptake of Ca^2+^ ions and prevents mitochondrial calcium overload [30]. Under resting cellular conditions, the uniporter is in a dormant state and is activated only when the local Ca^2+^ ion level increases to approximately 1 μM or more [31]. Halestrap et al. [32] reported that an increase in the mitochondrial Ca^2+^ concentration could increase the electrical flux of K^+^ ions, thereby leading to an increase in mitochondrial volume. However, many studies have provided different interpretations of this phenomenon.

In 1999, Siemen et al. [33] discovered a Ca^2+^-activated selective K^+^ channel (MitoKCa) in the mitochondrion of human brain glioma cells, and in 2002, Xu et al. [34] detected a channel in the mitochondrion of cardiomyocytes. O’Rourke et al. [35] suggested that MitoKCa may be activated under pathophysiological conditions to increase the uptake of mitochondrial Ca^2+^ ions, prevent excessive accumulation of mitochondrial Ca^2+^ ions and play a physiological role in fine-tuning mitochondrial volume or Ca^2+^ accumulation under increased cardiac load.

Studies have suggested that the activity of K^+^ efflux pathways may also be regulated by divalent cations such as Ca^2+^. Evidence indicates that depletion of endogenous divalent cations induces the activity of electroneutral K^+^/H^+^ antiporters [36,37], and Gogvadze et al. proposed that Ca^2+^ ions exert inhibitory effects on the activity of mitochondrial K^+^/H^+^ antiporters, and the uncompensated influx of K^+^ ions can promote net water flow and cause mitochondrial swelling [11]. In addition, when mitochondrial Ca^2+^ overload occurs, the pathway for Ca^2+^ efflux (Na^+^/Ca^2+^ exchange) is activated, leading to the loss of proton gradient. The loss of proton gradient inhibits the function of K^+^/H^+^ exchangers, finally resulting in mitochondrial swelling [2,38].

**Figure 2.**
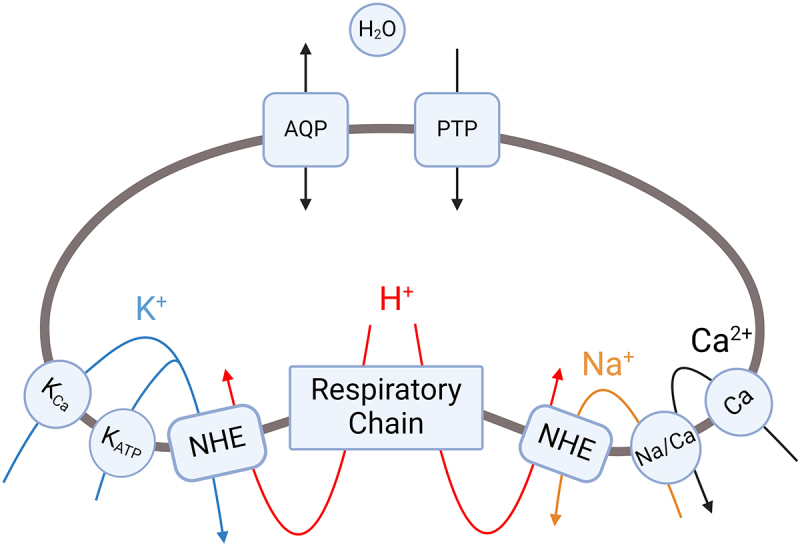
Cation fluxes that regulate mitochondrial matrix volume.

If the Na^+^/Ca^2+^ exchanger is saturated and mitochondrial Ca^2+^ concentration continues to increase, this can promote opening of the mitochondrial permeability transition pore (PTP) under pathological conditions [38]. Full opening of the PTP leads to mitochondrial permeability transition, a process of destructive mitochondrial swelling rather than a regulated volume change. However, under physiological conditions, the PTP can transiently open with low conductance, as described by Javadov et al. [39]. Low-conductance PTP opening increases the permeability of the inner mitochondrial membrane to solutes below 300 Da, primarily ions like K^+^. This selective ion flux can help regulate mitochondrial matrix volume through osmotic changes. The mechanisms by which Ca^2+^ can induce regulated changes in mitochondrial volume primarily involve the influx of K^+^ ions, which affects osmotic balance (Figure 2). However, excessive Ca^2+^ accumulation beyond buffering capacity can instead promote pathological PTP opening and mitochondrial permeability transition, resulting in mitochondrial destruction rather than a regulated volume change. In summary, while Ca^2+^ influx can modulate matrix volume through regulated K+ flux under physiological conditions, excessive Ca^2+^ overload instead promotes pathological PTP opening and mitochondrial permeability transition, leading to mitochondrial destruction rather than regulated volume changes.

K_Ca_, Ca^2+^-dependent K^+^ channel, KATP, ATP-dependent K^+^ channel, KHE, K^+^/H^+^ exchanger, NHE, Na^+^/H^+^ exchanger, Na/Ca, Na^+^/Ca^2+^ exchanger, AQP, aquaporin; PTP, permeability transition pore.

## Anions regulate mitochondrial volume homeostasis

### Anion channels in the mitochondrion

Similar to the anion channels in the plasma membrane, those in the mitochondrion are protein pores that allow anions to passively diffuse along their electrochemical gradient. According to their location, they can be divided into outer mitochondrial membrane anion channels and inner mitochondrial membrane anion channels (IMACs).

Voltage-dependent anion channels (VDACs) are the main anion channels located on the outer mitochondrial membrane and form the main route for the entry and exit of metabolites and ions in the membrane. They are widely distributed in many species, with two types in yeast (POR1 and POR2) and three subtypes in vertebrates (VDAC1, VDAC2 and VDAC3). VDACs are believed to mediate the release of cytochrome c, leading to apoptosis [40].

The understanding of IMACs is derived from early studies on mitochondrial swelling. IMACs are active under specific conditions (Mg^2+^ depletion, matrix alkalization, etc.) and are highly permeable to various inorganic anions (Cl^−^, NO_3_^−^, SCN^−^, Pi, etc.) and organic anions (succinate 2−, malate 2−, ATP4−, etc.) [41]. IMACs are mainly related to mitochondrial volume homeostasis and play a role in arrhythmia and contractile dysfunction after myocardial ischemia [35,42].

### Relationship between cell volume and mitochondrial volume mediated by chloride ions

Cl^−^ is the most abundant and common anion in the body that plays important roles in various physiological processes such as cellular excitability regulation, transmembrane substance transport, cell volume regulation and organelle acidification. Given that, under physiological conditions, ion transport mediated by anion channels is mainly Cl^−^, anion channels are commonly referred to as chloride channels [43]. Based on differences in structure or function, chloride channels can be divided into several categories: ligand-gated chloride channels, cystic fibrosis transmembrane conductance regulators (CFTRs), chloride intracellular channels (CLICs), voltage-gated chloride channels (CLCs) and Ca^2+^-activated chloride channels (ClCa) [44].

**Figure 3.**
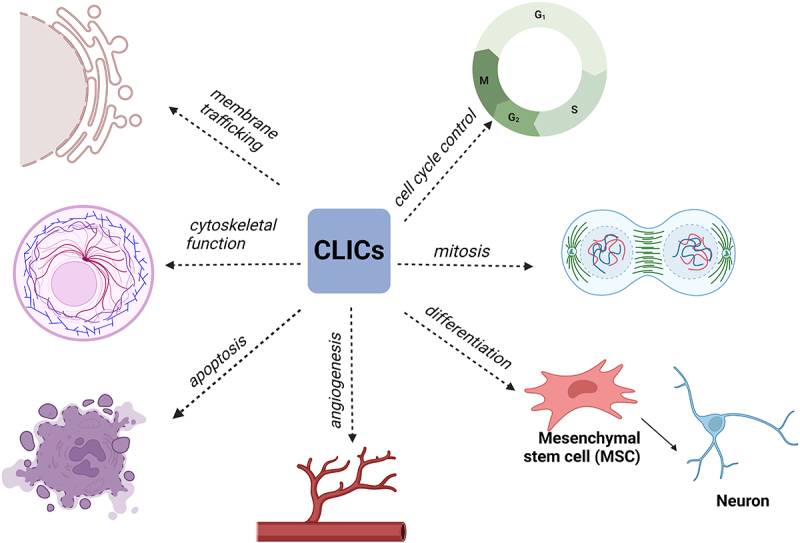
Regulation of chloride intracellular channels (CLICs).

CLICs are a newly discovered type of chloride channel, mainly located in the intracellular membrane. In mammals, six homologous anion channels (CLIC1–6) exist in the soluble or integral membrane protein form and have dual functions as enzymes and channels. As multifunctional proteins, they are involved in membrane transport, cell skeleton function, cell cycle regulation, tubular system generation, vascular endothelial cell generation, mitosis and differentiation (Figure 3). Thus far, the only CLIC proteins known to exist in the mitochondrial membrane are CLIC4 and CLIC5; CLIC4 is located in the outer mitochondrial membrane, also known as MtCLIC, whereas CLIC5 is located in the inner mitochondrial membrane [40].

CLICs are involved in membrane transport, cell skeleton function, cell cycle regulation, tubular system generation, vascular endothelial cell generation, mitosis and differentiation.

A notable feature of apoptosis is cell shrinkage, and the activation of ion channels is an essential process in regulating apoptosis. The anion channels drive the movement of water molecules by releasing anions and organic ions into the extracellular matrix, leading to cell shrinkage [45,46]. Cl^−^ is one of the main ions involved in cell volume regulation [47]. Several Cl^−^ ion channels on the cell membrane, such as LRRC8, anoctamin and CFTR, play important roles in regulating apoptosis [45]. During apoptosis, a large amount of Cl^−^ flows out into the extracellular matrix, which increases the ion gradient both inside and outside of the mitochondrion, stimulating the opening of CLIC on the inner mitochondrial membrane, thus promoting electrogenic Cl^−^ efflux from the mitochondrion and decreasing the mitochondrial volume. Tomaskova et al. [44] reported that the inner mitochondrial membrane potential disappears during apoptosis, which may activate voltage-dependent mtCl channels. Mitochondrial chloride ion channels regulate volume during apoptosis by providing Cl^−^ ions or by serving as potential regulators or counter ions for K^+^ efflux during apoptosis.

## Implications of imbalance in mitochondrial volume regulation

### Mitochondrial dynamics and volume homeostasis

Mitochondrial dynamics is a process in cells that refers to the constant division and fusion of the mitochondrion to provide energy for regular cellular processes and regulate processes such as autophagy, calcium homeostasis, innate immunity, signal transduction and apoptosis [48]. This process is regulated by two opposing processes: mitochondrial fusion and fission, mitochondrial synthesis and autophagy and intracellular transport. These processes maintain the dynamic balance in mitochondrial volume and regulate mitochondrial shape, volume and function and are increasingly recognized as a key component of the cellular stress response [49].

Mitochondrial fission separates the damaged mitochondrion from the maternal mitochondrion, which is conducive to autophagy to eliminate the damaged ones. By contrast, mitochondrial fusion combines dysfunctional mitochondrion with healthy ones and dilutes the damaged components to repair the damaged mitochondrion, thereby preventing the organelle from being cleared through autophagy [50]. Mitochondrial fission is usually predominant in cells under stress and those entering the death phase, and this process is closely related to mitochondrial dysfunction [51]. Mitochondrial fusion is usually predominant in the autophagy process induced by nutrient deficiency and increasingly occurs to prevent being engulfed during the autophagy process, and this process is mainly related to cell survival mechanisms. The relative balance in mitochondrial fission and fusion is crucial to maintaining the quality and function of the mitochondrion and is an important basis for ensuring normal cellular activities [52].

The protein that regulates mitochondrial fission in mammalian cells is mainly dynamin-related protein 1 (Drp1). Drp1 is necessary for mitochondrial fission. This protein is usually localized in the cytoplasm under physiological conditions and mainly comprises an N-terminal GTPase domain, a middle helical domain and a C-terminal GTPase effector domain [53,54].

Various stimuli promote the recruitment and oligomerisation of Drp1 to the mitochondrion by binding to its receptors, such as Mff [55,56], Fis1 [54], MiD49 and MiD51 [57–59]. Multiple Drp1 molecules tightly surround the mitochondrion to form a ring-like structure and rely on their GTPase activity to hydrolyze GTP, leading to the division of the inner and outer mitochondrial membranes and resulting in mitochondrial fission. Parkin is an upstream regulatory molecule of Drp1 and can mediate Drp1 degradation by proteasomes. If the Parkin level in the cell decreases, then the Drp1 degradation process will be inhibited, leading to an increase in Drp1 activity and excessive mitochondrial fission, finally leading to the occurrence of related diseases [60].

**Figure 4.**
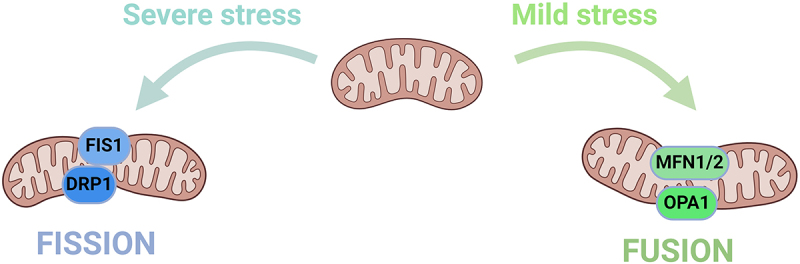
Mechanism of mitochondrial dynamics.

Mitochondrial fusion is mainly divided into several processes, including mitochondrial tethering, outer membrane fusion and inner membrane fusion, all of which are mediated by various proteins such as mitofusin 1 (Mfn1), mitofusin 2 (Mfn2) and optic atrophy 1 (OPA1) [61–63]. Mfn1 and Mfn2 are widely expressed in the outer mitochondrial membrane and mediate outer membrane fusion, whereas OPA1 mainly participates in inner membrane fusion. (Figure 4)

The mitochondrion is in a dynamic equilibrium of simultaneous fission and fusion, and this equilibrium is disrupted when the internal and external cellular environments change. When cells experience mild stress, the mitochondrion forms an elongated and interconnected network to resist mitochondrial autophagy and to increase ATP production, thereby adapting to stressful conditions such as cellular undernutrition. By contrast, under severe stress, the mitochondrion becomes fragmented. When the dynamics of mitochondrial fission and fusion become unbalanced, intracellular homeostasis is disturbed, leading to mitochondrial autophagy and even apoptosis.

The regulation of mitochondrial volume is a key issue in cellular pathophysiology. Mitochondrial volume and shape can undergo alterations after regulated fission-fusion, which is mediated by a complex network of cytoplasmic and mitochondrial proteins and involves ion transport across the inner mitochondrial membrane [64]. Any imbalance in the mitochondrial dynamics affects the steady state of mitochondrial volume.

### Imbalance in mitochondrial volume homeostasis and the subsequent apoptosis and autophagy

Regulated cell death is the basis for tissue development and maintenance through the elimination of unwanted cells. Inhibiting cell death can lead to the development of cancer and autoimmune diseases, while excessive cell death can lead to the development of neurodegenerative diseases including Parkinson’s disease, Alzheimer’s disease, amyotrophic lateral sclerosis and Huntington’s disease [65]. Apoptosis is a major form of cell death regulation and plays a central role in many processes, from embryonic development to immune homeostasis.

Cell apoptosis can occur through two pathways: extrinsic and intrinsic. The intrinsic pathway, also known as the mitochondrial pathway, is induced by several types of stimuli that activate BH3-only proteins in the B-cell lymphoma 2 (BCL-2) protein family. BH3-only proteins inhibit anti-apoptotic BCL-2 proteins and activate pro-apoptotic BCL-2-associated X protein (Bax) and BCL-2 antagonist/killer 1, leading to an increase in outer mitochondrial membrane permeability [65]. The increase in mitochondrial permeability leads to cell death through several mechanisms. The first is the release of mitochondrial proteins that are functional effectors of cell death in the cytoplasm. These effectors can be divided into two categories: some effectors induce programmed cell death by activating caspases (proteinases that execute cell destruction that is characteristic of programmed cell death), and others induce this process without relying on caspase activity, such as apoptosis-inducing factor. The second major mechanism is the loss of mitochondrial membrane potential caused by membrane permeability, which inhibits oxidative phosphorylation and reduces ATP synthesis, resulting in a bioenergetic catastrophe that leads to cell death [66].

Frank et al. [67]indicated that the dynamics of mitochondrial morphology play an important role in regulating cell apoptosis. Overexpression of the dominant negative mutated form of Drp1 that elongated mitochondria inhibits the release of cytochrome c and delays the apoptotic response. Jagasia et al. [68] pointed out that alterations in mitochondrial dynamics through Drp1 in some non-mammalian species can delay or even prevent cell apoptosis. Some studies have shown that depletion of OPA1 can lead to a reduction in mitochondrial fusion, while overexpression of FIS1 can increase mitochondrial division and can promote apoptosis [69–71]. These findings suggest that mitochondrial morphology and fusion and fission rates are key parameters that promote cell apoptosis. However, some studies have also demonstrated that an increase in the mitochondrial fission rate is not always related to the activation of cell apoptosis. For example, Mfn1−/− and Mfn2−/− cells exhibit widespread mitochondrial fragmentation but still survive completely [72], indicating that mitochondrial division based on the expression of mitochondrial dynamics genes is not always related to cell apoptosis. In summary, whether the mitochondrial division is a key factor for regulating cell apoptosis or just an accompanying phenomenon of programmed cell death remains an unresolved question.

Autophagy is a highly conserved lysosome-dependent cellular degradation process in eukaryotic cells, and it can be divided into several stages such as autophagy induction, nucleation and formation of isolation membranes, the extension of isolation membranes, closure of membranes to form autophagosomes, the fusion of autophagosomes and lysosomes and degradation of contents [73]. Mitochondrial autophagy refers to the selective wrapping and degradation of the damaged mitochondria in cells through autophagy mechanisms, which plays an important role in maintaining the stability of the mitochondrial network and the quality and quantity of the mitochondria [74].

Mitophagy is regulated through various pathways, broadly divided into two categories: ubiquitin-dependent mechanisms [75,76], and ubiquitin-independent mechanisms [77–85], both of which require LC3 adaptor proteins. It plays an important role in maintaining mitochondrial volume homeostasis including mitochondrial dynamics, and a mutual regulation exists between mitochondrial dynamics and mitophagy. For example, Drp1, an important mitochondrial fission protein, interacts with the LC3 receptor FUNDC1 and BCL2L13 to induce mitophagy [86]; the LC3 receptor cardiolipin, an inner mitochondrial membrane protein, mediates mitochondrial inner membrane fusion by binding to OPA1 [87]. This suggests that disruption of the steady-state mitochondrial dynamics – fission and fusion – leads to a decrease or increase in mitochondrial volume, which can affect mitophagy.

### Mams and mitochondrial volume

The distribution and dynamics of mitochondria are influenced by the physical connections between the outer mitochondrial membrane and various intracellular membranes such as the plasma membrane, peroxisomes, endoplasmic reticulum, autophagosomes and lysosomes, collectively known as mitochondria-associated membranes (MAMs) [88]. Among them, the connection between the endoplasmic reticulum and the mitochondria was the first identified inter-organelle contact site [89]. The structure of MAMs dynamically changes with alterations in cell status, and the gap width between the endoplasmic reticulum and the outer mitochondrial membrane ranges from 10 to 100 nm [90,91], usually 10–15 nm at the smooth endoplasmic reticulum and 20–30 nm at the rough endoplasmic reticulum, possibly attributed to the presence of ribosomes [92]. Different researchers have analyzed the structure of MAMs using different proteomic approaches and reported that 991 [93] and 1212 [94] different proteins exist in MAMs. Based on mass spectrometry analysis, these proteins have been classified into three categories: proteins specifically present in MAMs, proteins present in both MAMs and other organelle structures, and proteins present only temporarily in MAMs. These proteins are involved in a wide variety of processes such as structure maintenance, lipid synthesis, regulation of Ca^2+^ homeostasis, mitochondrial dynamics and apoptosis [95].

MAMs play an important role in regulating mitochondrial shape and dynamics. Proteins involved in regulating mitochondrial fusion and fission, such as DRP1 and MFN2, as well as proteins involved in mitochondrial movement, such as Rho-GTPases (Miro1 and Miro2), are enriched in MAMs [96]. The induction of mitochondrial fission at the endoplasmic reticulum – mitochondria contact site is well established, with the endoplasmic reticulum tubules wrapping around the mitochondria and marking the position for the recruitment of the mitochondrial fission receptor DNM1 (in yeast) or Drp1 (in mammals), which assemble to form a spiral to constrict the mitochondria [86]. Miro1 and Miro2, which are located on the outer mitochondrial membrane, have two Ca^2+^-binding domains and can sense high levels of Ca^2+^ to regulate mitochondrial movement [96]. These proteins play an important role in tethering the mitochondrion to the cytoskeleton by binding to motor proteins, thereby making mitochondrial movement dependent on cytosolic Ca^2+^ levels. At a high cytosolic Ca^2+^ concentration, Miro proteins dissociate from the motor proteins, inducing the cessation of mitochondrial movement. They also enhance mitochondrial fusion under resting cytoplasmic Ca^2+^ concentration but promote mitochondrial fragmentation under elevated concentrations of cytoplasmic Ca^2+^. Therefore, Miro proteins, which are Ca^2+^-sensitive regulatory factors, play a role in the dynamics of mitochondrial movement and fusion/fission [97].

IP3Rs are important Ca^2+^ efflux channels located on the surface of the endoplasmic reticulum and mediate the release of Ca^2+^ from the endoplasmic reticulum lumen to the cytoplasm [95]. Ca^2+^ released from the endoplasmic reticulum through IP3Rs then passes through the outer mitochondrial membrane through the Ca^2+^ permeable channel VDAC [98,99] and reaches the MCU located on the inner mitochondrial membrane, where it cooperatively regulates the influx of Ca^2+^ into the mitochondrial matrix [100]. Excessive accumulation of Ca^2+^ in the mitochondria activates the mitochondrial PTP, resulting in increased permeability of the inner mitochondrial membrane, dissipation of the mitochondrial membrane potential, termination of ATP synthesis, uncontrolled entry of water into the mitochondrial matrix and subsequent mitochondrial swelling, outer mitochondrial membrane rupture, cytochrome c release and cell apoptosis [101]. Thus, MAMs can regulate the fusion/fission mechanisms of the mitochondrion and mediate the flux of Ca^2+^ to affect the mitochondrial volume homeostasis.

### Related diseases caused by an imbalance in mitochondrial volume homeostasis

Mitochondrial volume homeostasis is influenced by multiple factors, and any imbalance in homeostasis is associated with the development of various diseases. Charcot-Marie-Tooth neuropathy type 2A (CMT2A) is a well-known genetic neurological disease affecting the peripheral nervous system, caused by mutations in the *Mfn2* gene [102,103]. These mutations are mostly located in or near its GTPase domain and mitochondrial targeting region, which are regions responsible for mitochondrial fusion [102,104]. In addition, OPA1 dysregulation can lead to susceptibility to dominant optic atrophy (an inherited optic neuropathy) [105]. Any imbalance in mitochondrial fission and fusion can also cause neurodegenerative diseases such as Parkinson’s disease caused by autosomal recessive mutations in the *PINK1* or *Parkin* gene, which result in abnormalities in the accumulation of small mitochondria and Drp1 [106,107]. In fibroblasts of patients with Huntington’s chorea, dysfunctional huntingtin protein interacts with Drp1, promoting its GTPase activity and causing unrestricted mitochondrial fission [108]. Apart from cardiomyopathy caused by excessive fission, the skeletal muscles of obese individuals and patients with type 2 diabetes also show small and round mitochondria [104,109,110]. Wu et al.‘s study [111] showed that Drp1 knockout can inhibit mitochondrial autophagy in cells and consequently suppress apoptosis of retinal endothelial cells, thus alleviating diabetic retinopathy. Deng et al. [112] suggested that mitochondrial volume homeostasis may be involved in the homeostasis of cartilage matrix metabolism and play an important role in the progression of arthritis.

## Summary

In recent years, homeostasis in mitochondrial dynamics has received widespread attention. The normal morphology and function of the mitochondrion is an important requirement for ensuring various physiological activities in the cell, as it is an important organelle. Mitochondrial dysfunction is a common feature associated with many diseases such as neurodegenerative diseases and cardiovascular diseases. Therefore, mitochondrial dynamics has become a potentially effective therapeutic target for treating such major diseases. Our review elaborated on the relevant regulatory mechanisms underlying mitochondrial fission, fusion, and dynamic balance, and we hope that, based on these findings, small-molecule compounds targeting various regulatory proteins could be developed to maintain mitochondrial volume homeostasis and to achieve effective treatment. Further in-depth exploration of the mechanisms involved in maintaining mitochondrial homeostasis and regulating mitochondrial autophagy will provide more insights into the molecular mechanisms underlying the occurrence of various diseases and will lay a theoretical foundation for the development of new drugs targeting mitochondrial dynamics proteins or mitochondrial autophagy regulatory proteins. Although significant progress has been made in this field of research, the interpretation of fission-fusion imbalance may not be accurate enough for understanding the various pathological symptoms of mitochondrial dysfunction – related diseases. More evidence is needed to understand mitochondrial behavior and to connect mitochondrial dynamics with other behaviors (such as its quality control pathways), which will fundamentally help adopt a more comprehensive perspective on the treatment and intervention of mitochondrial diseases.

## Data Availability

Data sharing is not applicable to this article as no new data were created or analyzed in this study.
